# Reduced expression of miRNAs as potential biomarkers in axial spondyloarthritis

**DOI:** 10.1590/1806-9282.20231521

**Published:** 2024-05-03

**Authors:** Nurdan Oruçoğlu Yıldırım, Şenay Balcı, Lülüfer Tamer

**Affiliations:** 1 Mersin University, Faculty of Medicine, Department of Internal Medicine, Division of Rheumatology – Mersin, Turkey.; 2 Mersin University, Faculty of Medicine, Department of Medical Biochemistry – Mersin, Turkey.

**Keywords:** Axial spondyloarthritis, microRNA, Uveitis

## Abstract

**OBJECTIVE:**

This study aimed to investigate the value of miR-29a-3p, miR-27a, miR126-3p, miR-146a-5p, miR-625-3p, miR-130a, miR-32, miR-218, miR-131, and miR5196 in the diagnosis of axial spondyloarthritis and to determine whether there is a difference in miRNA expression levels between radiographic axial spondyloarthritis and non-radiographic axial spondyloarthritis, as well as the relationship between miRNA expression levels, disease activity, and uveitis history.

**METHODS:**

This study included 50 patients with axial spondyloarthritis (25 with radiographic axial spondyloarthritis and 25 with non-radiographic axial spondyloarthritis) and 25 healthy individuals. The fold change of miRNA expression for each miRNA was calculated using the 2^-ΔΔCt^ method.

**RESULTS:**

The expression of all miRNAs except miR-130a was downregulated in axial spondyloarthritis patients (miR-27a: fold regulation: −11.21, p<0.001; miR-29a-3p: fold regulation: −2.63, p<0.001; miR-32: fold regulation: −2.94, p=0.002; miR-126-3p: fold regulation −10.94, p<0.001; miR-132: fold regulation: −2.18, p<0.001; miR-146-5p: fold regulation: −9.78, p<0.001; miR-218: fold regulation: −2.65, p<0.001; miR-625-3p: fold regulation: −2.01, p=0.001; miR-5196-3p: fold regulation: −7.04, p<0.001). The expression levels of these miRNAs did not differ significantly between non-radiographic axial spondyloarthritis and radiographic axial spondyloarthritis patients (p>0.05 for all).

**CONCLUSION:**

Particularly, miR-27a, miR-126-3p, miR-146-5p, and miR-5196-3p were found to be substantially downregulated in both non-radiographic axial spondyloarthritis and radiographic axial spondyloarthritis patients, suggesting their potential as diagnostic biomarkers for axial spondyloarthritis.

## INTRODUCTION

Axial spondyloarthritis (AxSpA) is a chronic inflammatory disease that mainly involves the axial joint and significantly affects the quality of life and functionality^1^. Although the exact pathogenesis of AxSpA remains elusive, many genetic, environmental, and immunological factors contribute to its pathogenesis^2^.

MiRNAs are known to have a role in various processes, including the regulation of inflammation, bone resorption, and formation in AxSpA^3^. There is still debate about whether non-radiographic AxSpA (nr-AxSpA) should be considered a distinct condition from radiographic AxSpA (r-AxSpA) or an early stage of r-AxSpA. Although nr-AxSpA resembles r-AxSpA regarding disease activity, health status, and functionality, it differs from nr-AxSpA in the degree of inflammation and the lack of structural damage in most patients, even after many years^4^. This suggests that nr-AxSpA may have a distinct miRNA expression profile. Despite the increasing amount of research on miRNA expression in r-AxSpA patients, few studies have focused on the difference between miRNA profiles of nr-AxSpA and r-AxSpA^5^.

The aim of this study was to investigate the expression levels of target 10 miRNAs in AxSpA patients and determine whether there was a difference between r-AxSpA and nr-AxSpA. Furthermore, the association between miRNA expression levels and the presence of uveitis history and the correlation between inflammatory markers and disease activity was also evaluated.

## METHODS

### Study participants

This cross-sectional study included a total of 50 patients diagnosed with AxSpA (25 with nr-AxSpA and 25 with r-AxSpA) and 25 age and gender-matched healthy controls (HCs). The study only included participants who were naïve to biological treatments. Healthcare professionals who visited the general internal medicine outpatient clinic for periodic control and had no known diseases comprised the HC group.

The Bath Ankylosing Spondylitis Disease Activity Index (BASDAI) and the Ankylosing Spondylitis Disease Activity Score-C-reactive protein (ASDAS-CRP) scores were used to assess the disease activity of AxSpA patients.

The Institutional Clinical Research Ethics Committee of Mersin University approved the study (Approval No: 2019/322, Date: 24/07/2019). All participants provided informed, written consent before the study.

### miRNA analysis

For miRNA analysis, the samples were centrifuged at 2,000 *g* for 10 min without waiting for more than 2 h at room temperature. The aliquoted plasma samples were stored at −80°C in a freezer. MiRNA isolation was performed from plasma samples using a miRNA isolation kit (Roche Diagnostics, GmbH, Mannheim, Germany). After isolation, the samples were converted to cDNA and stored at −20°C until the analysis. The expression analysis of the 10 target miRNAs was performed using a real-time polymerase chain reaction (RT-PCR) device (Roche LightCycler 480). The Ct values of the miRNAs were normalized using the reference RNU6B. The fold change of miRNA expression for each miRNA was calculated as the 2^-ΔΔCt^ method^6^.

### Statistical analysis

The statistical software program SPSS (Statistical Package for Social Sciences) version 22.0 for Windows (IBM Inc., Chicago, IL, USA) was used to analyze the data. To test the normal distribution of data, the Shapiro-Wilk test was used. Independent-samples Student's t-test or Mann-Whitney U test was used to compare the two groups. Comparisons between more than two groups were performed using the analysis of variance (ANOVA) test or the Kruskal-Wallis test. For the correlation between the parameters, Pearson's or Spearman's correlation test was used. Receiver operating characteristics (ROC) analysis was used to evaluate the potential of each miRNA to differentiate between AxSpA patients and HCs. p<0.05 was considered statistically significant.

## RESULTS

The demographic characteristics and clinical and laboratory data of 25 nr-AxSpA, 25 r-AxSpA, and 25 HCs are presented in Table 1.

**Table 1:** Differences between demographic characteristics, laboratory data, and disease activity scores of axial spondyloarthritis patients and healthy controls.

	nr-AxSpA (n=25) (mean+SD)	r-AxSpA (n=25) (mean+SD)	HC (n=25) (mean+SD)	p-value
Age (years)	39.72±6.71	41.68±9.99	40.55±6.43	0.664
Sex (n/%) (female/male)	14/11 (56/44%)	10/15 (40/60%)	12/13 (48/52%)	0.399
ESR, mm/h	16.12±9.97	19.22±16.62	5.42±4.08	<0.001†
				<0.001‡
				0.922§
CRP, mg/L	9.06±10.75	13.39±13.39	2.26±1.65	<0.001†
				<0.001‡
				0.265§
Disease duration (months)	51.76±35.78	115.2±103.53	N/A	0.032
HLA-B27 positivity (n/%)	13 (52%)	17 (68%)	N/A	0.253
BASDAI	3.79±1.77	4.26±1.20	N/A	0.317
ASDAS-CRP	2.85±1.13	3.29±0.89	N/A	0.133

SD: standard deviation; N/A: not assessed;

† p-value between HC and Nr-AxSpA;

‡ p-value between HC and r-AxSpA;

§p-value between nr-AxSpA and r-AxSpA. p<0.05 is considered statistically significant.

The expression levels of 10 miRNAs in nr-AxSpA and r-AxSpA patients and HCs and the differences between the groups are presented in Table 2.

**Table 2:** Expression levels of plasma miRNAs in patients with non-radiographic axial spondyloarthritis, radiographic axial spondyloarthritis, and healthy controls.

	HC ΔCt (mean±SD)	nr-AxSpA ΔCt (mean±SD)	r-AxSpA ΔCt (mean±SD)	p-value
miR-27a	1.88±1.69	5.99±2.49	5.50±1.54	<0.001†
				<0.001‡
				0.649§
miR-29a-3p	5.93±1.35	8.42±2.01	7.23±1.60	<0.001†
				0.009‡
				0.066§
miR-32	2.97±1.82	5.06±25	4.60±1.70	0.001†
				0.011‡
				0.684§
miR-126-3p	0.94±1.85	5.19±2.59	4.42±1.86	<0.001†
				<0.001‡
				0.204§
miR-130a	-2.02±1.73	-0.71±2.44	-2.18±1.95	0.06
miR-132	3.06±1.38	5.75±2.55	5.07±1.69	<0.001†
				<0.001‡
				0.510§
miR-146-5p	-3.18±4.57	4.57±3.24	3.07±2.58	<0.001†
				<0.001‡
				0.177§
miR-218	-4.93±1.37	-2.39±2.39	-3.20±2.20	<0.001‡
				0.005‡
				0.438§
miR-625-3p	2.43±2.94	5.13±2.78	4.67±2.15	0.005†
				0.010‡
				0.789§
miR-5196-3p	4.02±1.92	6.81±1.52	6.55±1.64	<0.001†
				<0.001‡
				0.859§

ΔCt: delta cycle threshold; SD: standard deviation;

† p-value between HC and Nr-AxSpA;

‡ p-value between HC and r-AxSpA;

§p-value between nr-AxSpA and r-AxSpA. p<0.05 is considered statistically significant.

No significant correlation was found between miRNA expression levels and CRP, ESR, BASDAI, and ASDAS-CRP levels of AxSpA patients for any of the 10 miRNAs (p>0.05 for all parameters).

The expression of all miRNAs except miR-130a was downregulated in all AxSpA patients [miR-27a: Fold Regulation (FR): −11.21, p<0.001; miR-29a-3p: FR: −2.63, p<0.001; miR-32: FR: −2.94, p=0.002; miR-126-3p: FR: −10.94, p<0.001; miR-132: FR: −2.18, p<0.001; miR-146-5p: FR: −9.78, p<0.001; miR-218: FR: −2.65, p<0.001; miR-625-3p: FR: −2.01, p=0.001; miR-5196-3p: FR: −7.04, p<0.001]. miR-130a expression was not different between HCs and AxSpA patients (FR: −1.14, p=0.402). The expression levels of these nine miRNAs did not differ significantly between nr-AxSpA and r-AxSpA patients (p>0.05, for all).

Table 3 presents AUC, sensitivity, specificity, and optimal cutoff values for 10 miRNAs.

**Table 3:** Receiver operating characteristics analysis for target miRNAs to diagnose axial spondyloarthritis.

	AUC	95%CI	Optimal cutoff	Sensitivity	Specificity	p-value
miR-27a	0.933	0.866–0.999	3.57	90%	88%	<0.001
miR-29a-3p	0.828	0.724–0.931	6.43	78%	84%	<0.001
miR-32	0.756	0.639–0.872	3.79	70%	76%	<0.001
miR-126-3p	0.892	0.819–0.965	2.6	74%	76%	<0.001
miR-132	0.836	0.740–0.931	3.65	76%	80%	<0.001
miR-146-5p	0.962	0.914–1.000	0.24	94%	92%	<0.001
miR-218	0.816	0.713–0.920	-4.46	74%	76%	<0.001
miR-625-3p	0.749	0.637–0.860	3.05	72%	68%	<0.001
miR-5196-3p	0.846	0.742–0.949	5.33	78%	80%	<0.001

AUC: area under curve; CI: confidence interval; statistically significant at p<0.05.

The expressions of miR-29a-3p and miR-146-5p were significantly lower in AxSpA patients with a history of uveitis compared with those without uveitis (miR-29a-3p: FR: −2.56, p=0.001 and miR-146-5p: FR: −2.56, p=0.034, respectively).

## DISCUSSION

Our results revealed that miR-27a, miR-29a-3p, miR-32, miR-126-3p, miR-132, miR-146-5p, miR-218, miR-625-3p, and miR-5196-3p were found to be significantly downregulated in AxSpA patients compared with healthy individuals and might be a promising diagnostic biomarker for AxSpA. MiR-29a-3p and miR-146-5p were also associated with a history of uveitis in AxSpA patients. Thus, these miRNAs may play a role in the pathophysiology of AxSpA and clinical features.

The progression of immune dysfunction and autoimmunity appears to be highly associated with changes in miRNA regulation^7^. Profiles of microRNA expression in AxSpA may serve as biomarkers for pathogenesis, diagnosis, prognosis, or treatment monitoring.

Despite nr-AxSpA and r-AxSpA being similar in terms of clinical presentation and disease activity, there is ongoing controversy as to whether nr-AxSpA represents a distinct disease entity or an early stage of ankylosing spondylitis (AS)^8^. The identification of the miRNA expression signatures of both forms of the disease, as well as the similarities and variances in expression levels, may give further objective evidence for points of view on this issue.

Diagnosis of patients in the non-radiographic stage can be challenging due to the lack of specific diagnostic tests for the disease, the fact that currently used tests such as ESR and CRP can be normal in some patients, and the high cost and limited accessibility of magnetic resonance imaging^9^. MiRNAs, which are known to have a role in the pathogenesis of the disease, may have the potential to serve as diagnostic biomarkers for AxSpA.

The same miRNA tested for an identical disease may produce conflicting results. This is assumed to be due to the fact that various factors, such as the origin of tissue samples, ethnic variations, and disease severity, may influence miRNA expression differently^10^. For instance, in some studies, miR-146 expression has been shown to be upregulated in AS patients. Chen et al.^11^ found no statistically significant difference between the expression levels of miR-146a-5p in Th17 cells of HCs and AS patients. In contrast, Fogel et al.^12^ reported downregulation of miR-146a-5p in the monocytes of AxSpA patients and a negative correlation between its level and ASDAS and CRP. Similarly, miR-146-5p was substantially downregulated in our study compared with HC in AxSpA patients. However, no correlation was found between miR-146-5p expression levels and disease activity or acute phase reactants.

In a study comparing the expression of miR-625-3p and miR-29a-3p in patients with nr-axSpA, AS, and HC, lower levels of expression were found in patients with AS than in patients with nr-AxSpA and HCs. However, in patients with nr-AxSpA, only miR-625-3p showed lower expression levels than in HC. miR-29a-3p expression levels were reported to be similar in patients with nr-AxSpA and HCs. The authors attributed this inverse relationship between radiographic bone formation and circulating miRNA levels of miR-29a-3p to its association with bone formation^13^. However, in our study, miR-625-3p and miR-29a-3p showed similarly low expression in both nr-AxSpA and r-AxSpA patients compared with HCs. Although these results suggest that miR-29a-3p may indicate the risk of radiographic progression in patients with nr-AxSpA, studies evaluating disease progression in these patients over time are needed.

Jiang et al.^14^ examined the relationship between miRNA-130a and its targets tumor necrosis factor (TNF)-1α and histone deactylase (HDAC)3 in AS patients’ peripheral blood mononuclear cells. Decreased miRNA-130a levels and increased HDAC3 and TNF-1 were observed in AS patients. However, miR-130a expression in AxSpA patients was similar to that in the healthy population in our study.

The most prevalent extra-articular manifestation of AxSpA is uveitis. Recurrent uveitis is a significant cause of morbidity that may lead to synechia and vision loss. No biomarker has yet been identified to predict the development of uveitis. Recent studies have indicated that a subset of microRNAs may serve as a biomarker for uveitis.

miR-146a, miR-146a-5p, miR-155, miR-182, and miR-223-3p were identified as potential biomarkers for uveitis in a systematic review^15^. In our study, miR29a-3p and miR-146-5p were found to be associated with uveitis.

The small sample size and single-center study are the main limitations of this study. As biological treatment-naive patients were included, which are substantially effective inhibitors of inflammation, the effect of these agents on miRNA signature could not be evaluated. Furthermore, patients were categorized only regarding sacroiliac joint involvement, and spinal involvement was not analyzed. In addition, the effect of geographic and ethnic differences on the expression profiles of miRNAs and the effect of disease progression over time on miRNA expression in nr-AxSpA patients were not evaluated in this study.

## CONCLUSION

Our study showed that especially miR-27a, miR-126-3p, miR-146-5p, and miR-5196-3p were significantly downregulated in both nr-AxSpA and r-AxSpA patients and had a high predictive value for AxSpA. It suggests that they have the potential to be used as a biomarker for the diagnosis of AxSpA. Furthermore, the similarity of all miRNA expression levels in individuals with nr-AxSpA and r-AxSpA supports the notion that these two disease types are the same disease entity. Additionally, lower expression of miR-29a-3p and miR-146-5p was associated with uveitis history in AxSpA patients. Investigating the expression and function of miRNAs in AxSpA may provide new insights into the underlying pathophysiology of AxSpA and could lead to the development of novel diagnostic biomarkers and treatment strategies.
